# Oxidative profile and protease regulator potential to predict sperm functionality in donkey (*Equus asinus*)

**DOI:** 10.1038/s41598-021-99972-9

**Published:** 2021-10-15

**Authors:** Stefano Cecchini Gualandi, Brunella Giangaspero, Tommaso Di Palma, Giuseppe Macchia, Augusto Carluccio, Raffaele Boni

**Affiliations:** 1 Department of Sciences, University of Basilicata, Campus Macchia Romana, 85100 Potenza, Italy; 2 Faculty of Veterinary Medicine, University of Teramo, Loc. Piano d’Accio, 64100 Teramo, Italy

**Keywords:** Reproductive biology, Assay systems, Spermatogenesis

## Abstract

Seminal plasma (SP) of donkey stallions was evaluated using various oxidative stress parameters as well as protease and protease inhibitor activities. SP was obtained by nine donkey stallions. In addition, one donkey stallion with non-obstructive azoospermia was enrolled in this study. Free radical scavenging activity (FRSA), the ferric reducing ability of plasma (FRAP), total antioxidant capacity (TAC), and total thiol level (TTL) were highly correlated with each other and with the protease inhibitor activity. However, only FRAP, TAC, and the nitrate/nitrite concentration (NO_x_) were significantly correlated with sperm concentration, production, and kinetics. Protease inhibitor activity was highly correlated with sperm concentration and production; however, it did not correlate with sperm kinetics. The azoospermic stallion produced a lower amount of semen than the normospermic stallions and its SP showed a lower antioxidant activity when evaluated with FRAP, TAC, and TTL as well as a higher NO_x_ and a lower protease inhibitor activity. In conclusion, the evaluation of SP oxidative profile by FRAP, TAC, and NO_x_ may provide reliable information on donkey sperm quality whereas protease inhibitor activity may play a role as a marker of the sperm concentration in this species.

## Introduction

Once produced in the testicle, the spermatozoa are conveyed along the male genital tract within a modified ultrafiltrate of the blood. At ejaculation, this fluid is enriched with the secretion of the accessory sexual glands. The resulting liquid matrix, the seminal plasma (SP), carries the sperm from the male into the female genital tract and plays relevant roles by preserving sperm functions, interacting with the female genital tract and modulating immune response as well as by promoting fertilization and successful pregnancy outcomes^1,2^,. The molecular composition of SP is species-dependent and represented by lipids, glycans, inorganic ions, and small molecule metabolites to biopolymers, such as cell-free DNA, RNA, microRNAs, peptides, proteins, and oligosaccharides^3,4^. Following the sperm placing into the female genital tract, the spermatozoa leave the SP for carrying out their procreative function and escape the female immune defenses appointed to counter the entry of foreign components. In mares, spermatozoa have been found in the oviduct within 1 h after insemination, and reach the fertilization site within 4 h after breeding^5^. Despite the very brief interaction between SP and spermatozoa, many functions are attributed to the SP that are important for reproductive activity and can be traced back as involving both the spermatozoa and the female genital tract. In stallion spermatozoa, SP functions to (i) provide metabolic and energetic compounds^6^; (ii) enhance sperm transport^7,8^; (iii) protect spermatozoa in breeding-induced endometritis from being phagocytosed and destroyed in an inflammatory environment^9^. In the female genital tract, SP functions to (i) provide an alkaline environment, thanks to basic polyamines, such as spermine, spermidine, and putrescine, that is useful to counteract the uterine acid environment^10^; (ii) hasten ovulation^11^; (iii) increase blood flow to the uterus and oviducts^12^; (iv) modulate the immune system as reducing the duration of the breeding-induced endometritis through suppressive effects on complement activation, polymorphonucleate (PMN)-chemotaxis, and phagocytosis^9^. Besides these positive functions, a recent study in donkey demonstrated a role of fresh and frozen SP in the occurrence of neutrophil extracellular traps (NETs) that are involved in the uterine immune defense against either infectious agents or spermatozoa with potential adverse effects on reproductive function^13^.

However, the activity of the SP takes on a different meaning when the semen is collected, manipulated, and stored, thus deferring the timing of the insemination. Among the properties attributable to SP and useful for out-of-body semen preservation, the ability to counteract the oxidative damage to which spermatozoa are subject is undoubtedly included^14^. Sperm cells are highly sensitive to oxidative damage^15,16^ due to their: (i) intense metabolic activity connected to movement^17^; (ii) low cytoplasmic component and, therefore, to the inability to accumulate intracellular compounds capable of counteracting oxidative damage^14,18^; (iii) high proportion of plasma membrane in relation to the cell mass containing a large quota of unsaturated fatty acids ensuring the membrane fluidity required for flagellar movement^19^; (iv) low content of DNA repair mechanisms^20^. The oxidative stress (OS) affects sperm quality and brings to DNA fragmentation, lipid membrane peroxidation, lowering motility, and, ultimately, in the reduction of fertility^16^. To assess the degree of seminal impairment from oxidative damage and the ability of seminal plasma to counteract it, numerous approaches have been used in humans and animal species^21,22^.

Besides OS, protease activity (PA) represents a further threat affecting semen quality. Proteolytic enzymes, as acrosin and hyaluronidase, are contained within the sperm acrosome as inactive precursors^23^. They are activated following their release during fertilization as well as from dead and damaged spermatozoa, and, hence, the assessment of their activity represents a sperm quality assay^24^. To counter this danger, SP contains protease inhibitors^25^.

This study was aimed to detect the oxidative profile as well as the protease and anti-protease activities of SP in donkey stallions. Both evaluations, here analyzed for the first time in the asinine species, are of relevant diagnostic interest as alterations in the oxidative and proteolytic balance have been associated with sperm dysfunctions in humans and other animal species^16,26^. The oxidative profile has been tested by evaluating the substances resulting from oxidative and nitrosative processes, as lipid hydroperoxide levels (total oxidant status, TOS) and nitrite/nitrate contents (nitric oxide radical metabolites, NO_x_), as well as the total antioxidant status, assessing the ferric reducing ability of plasma (FRAP), the total antioxidant capacity (TAC by ABTS-based assay), the total thiol levels (TTL) and the free radical scavenging activity (FRSA). Moreover, the advanced oxidation protein products (AOPP) were assessed as a marker of oxidative protein damage. Total proteins (TP), protease (PA) and antiprotease (APA) activities were assessed as biomarkers of proteolysis regulators. The results of these assays have been related to either semen quality or sperm kinetics. Finally, having identified an azoospermic individual within the group of animals trained for semen collection, it seemed interesting to compare the oxidative profile and the proteolytic regulatory potential of this individual with the rest of the normospermic group.

## Results

### Data report

Means (± SD) and 25–75% range of the oxidative profile and protease regulator parameters evaluated in the SP of the nine normospermic donkey stallions are showed in Table 1. In Table 2, there are the mean (± SD) values of the oxidative profile and protease regulator parameters assessed in the SP of the azoospermic stallion. Considering the availability of only one azoospermic stallion, it was not possible to perform any statistical comparison. However, several differences discriminated this azoospermic individual from normospermic stallions concerning the various methodologies used to evaluate the SP oxidative profile. In particular, the azoospermic stallion showed lower FRAP (83.9 ± 28.5 vs*.* 537.1 ± 184.0), FRSA (5.7 ± 2.4 *vs.* 22.6 ± 14.4), and TTL (53.4 ± 2.2 *vs.* 114.4 ± 36.8) values; vice versa, NO_x_ values were higher in azoo- than normo-spermic stallions (621.9 ± 7.7 vs*.* 216.7 ± 120.8). However, TAC, TOS, and AOPP were not detected in the azoospermic stallion. In addition, the SP of the azoospermic stallion showed a higher pH, a lower TP concentration, APA, and semen volume whereas the PA and osmolarity in the azoospermic stallion seem similar to those of the normospermic stallions.

**Table 1:** Mean (± SD) values and 25th–75th range of seminal plasma oxidative profile and proteolysis regulator parameters, semen parameters and sperm kinetics in normospermic donkey stallions (n = 9).

	Mean ± SD	25th–75th % range
**Seminal plasma parameters**		
FRAP (FeSO_4_·7H_2_O equivalents, μM)	537.1 ± 184.0	400.3–630.6
TAC (ascorbic acid equivalents, μM)	719.7 ± 254.3	488.6–918.3
FRSA (absorbance decrease, %)	22.6 ± 14.4	11.7–34.3
TTL (µM)	114.4 ± 36.8	88.7–147.2
TOS (*t*-BHP equivalents, μM)	2.1 ± 2.0	0.3–2.7
NOx (NaNO_3_ equivalents, μM)	216.7 ± 120.8	122.1–316.5
AOPP (chloramine-T equivalents, μM)	15.8 ± 9.6	11.3–24.3
PA (as bovine trypsin positive control, %)	32.4 ± 21.0	16.3–57.6
APA (trypsin activity inhibition, %)	69.5 ± 31.4	48.9–91.6
TP (BSA equivalents, mg mL^−1^)	55.4 ± 30.9	28.2–85.1
**Semen parameters and sperm kinetics**		
Osmolarity (mOsm)	293.9 ± 14.7	284.3–300.5
pH	7.64 ± 0.34	7.46–7.76
Volume (mL)	48.2 ± 16.9	31.8–55.0
Sperm concentration (10^6^ mL^−1^)	265.1 ± 96.0	201.4–335.3
Sperm production (10^9^)	12.5 ± 5.9	6.8–18.5
Tot mot (%)	75.8 ± 19.3	61.0–94.4
Prog (%)	27.9 ± 9.4	19.3–38.4
VCL (µm sec^−1^)	224.8 ± 25.5	205.0–242.7
VSL (µm sec^−1^)	82.1 ± 15.0	72.6–97.1
VAP (µm sec^−1^)	116.8 ± 22.3	95.8–138.6

FRAP: ferric reducing ability of plasma; TAC: total antioxidant capacity; FRSA: free radical scavenging activity; TTL: total thiol levels; TOS: total oxidant status; NOx: nitric oxide metabolites; AOPP: advanced oxidation protein products; PA: protease activity; APA: antiprotease activity; TP: total protein; Tot mot: total sperm motility; Prog: progressive sperm motility; VCL: curvilinear velocity; VSL: straight-line velocity; VAP: average path velocity.

**Table 2:** Mean (± SD) values of seminal plasma oxidative profile and proteolysis regulator parameters, and semen parameters in the azoospermic donkey stallion. Semen collections (n = 2).

	Mean ± SD
**Seminal plasma parameters**	
FRAP (FeSO_4_·7H_2_O equivalents, μM)	83.9 ± 28.5
TAC (ascorbic acid equivalents, μM)	0.0
FRSA (absorbance decrease, %)	5.7 ± 2.4
TTL (µM)	53.4 ± 2.2
TOS (*t*-BHP equivalents, μM)	0.0
NOx (NaNO_3_ equivalents, μM)	621.9 ± 7.7
AOPP (chloramine-T equivalents, μM)	0.0
PA (as bovine trypsin positive control, %)	20.6 ± 1.8
APA (trypsin activity inhibition, %)	3.6 ± 5.2
TP (BSA equivalents, mg mL^−1^)	4.2 ± 1.6
**Semen parameters**	
Osmolarity (mOsm)	293.0 ± 8.5
pH	8.45 ± 0.06
Volume (mL)	17.5 ± 3.5

FRAP: ferric reducing ability of plasma; TAC: total antioxidant capacity; FRSA: free radical scavenging activity; TTL: total thiol levels; TOS: total oxidant status; NOx: nitric oxide metabolites; AOPP: advanced oxidation protein products; PA: protease activity; APA: antiprotease activity; TP: total protein.

### Correlations between oxidative profile and protease regulator parameters

In Table 3, the correlation coefficients obtained by comparing each oxidative profile and proteolysis regulator parameter to each other are shown. Almost all the parameters were well associated with each other. However, TOS values were not significantly correlated with any of the other parameters analyzed whereas NO_x_ values were negatively correlated with FRAP and TAC and showed tendential associations with the other parameters. FRAP, FRSA, TAC, and TTL were correlated with the other parameters including those related to proteolysis regulators (PA and APA). AOPP was well correlated with FRSA, and TTL as well as with TP content, PA, and APA.

**Table 3:** Correlation matrix between oxidative profile and proteolysis regulator parameters in the seminal plasma of donkey stallions (n = 9).

	TAC	FRSA	TTL	TOS	NOx	AOPP	PA	APA	TP
FRAP	+ 0.934***	+ 0.580^†^	+ 0.697*	–	−0.738**	–	–	+ 0.775**	+ 0.684*
TAC		+ 0.654*	+ 0.787**	–	−0.809**	+ 0.630^†^	–	+ 0.780**	+ 0.746**
FRSA			+ 0.928***	–	–	+ 0.808**	+ 0.861**	+ 0.927***	+ 0.879***
TTL				–	−0.527^†^	+ 0.838**	+ 0.720*	+ 0.938***	+ 0.934***
TOS					–	–	–	–	–
NOx						−0.596^†^	–	−0.558^†^	−0.513^†^
AOPP							+ 0.727*	+ 0.867***	+ 0.894***
PA								+ 0.749*	+ 0.768*
APA									+ 0.917***

FRAP: ferric reducing ability of plasma; TAC: total antioxidant capacity; FRSA: free radical scavenging activity; TTL: total thiol levels; TOS: total oxidant status; NOx: nitric oxide metabolites; AOPP: advanced oxidation protein products; PA: protease activity; APA: antiprotease activity; TP: total protein. *, **, ***: indicate a statistic significance for P < 0.05, P < 0.01 and P < 0.001, respectively; ^†^: indicate a tendency for P < 0.10.

APA and the TP content practically correlated with all the other parameters except TOS and NO_x_, although for the latter the correlations are close to the cut-off value. PA was significantly correlated with FRSA, TTL, and AOPP as well as with TP content and APA.

### Correlations between oxidative and protease parameters *vs.* semen and sperm kinematic parameters

Table 4 shows the correlation coefficients between OS parameters and protease/proteases inhibitor activities *vs.* seminal and sperm kinematic parameters. Among the seminal parameters, the osmolarity and pH were not associated with any of the parameters analyzed, the seminal volume was only significantly associated with TOS and NOx values whereas the sperm concentration and production were significantly correlated with almost all the OS parameters, TP content, and APA but not related to TOS and PA. The sperm kinematic parameters (Tot mot, Prog, VCL, VSL, and VAP) were significantly correlated to FRAP, TAC, NO_x_ whereas AOPP was only correlated with total motility.

**Table 4:** Correlation matrix between oxidative profile and proteolysis regulator parameters in the seminal plasma, semen parameters and sperm kinetics in donkey stallions (n = 9).

	Osm	Vol	Conc	Prod	Tot mot	Prog	VCL	VSL	VAP
FRAP	–	–	+ 0.811**	+ 0.738**	+ 0.686*	+ 0.684*	+ 0.651*	+ 0.629*	+ 0.591^†^
TAC	–	+ 0.578^†^	+ 0.810**	+ 0.787**	+ 0.785**	+ 0.704*	+ 0.737**	+ 0.640*	+ 0.647*
FRSA	−0.524^†^	–	+ 0.873***	+ 0.824**	–	–	–	–	–
TTL	–	–	+ 0.905***	+ 0.864***	–	–	+ 0.510^†^	–	–
TOS	–	+ 0.822**	–	–	–	–	–	–	–
NOx	–	−0.745**	−0.636*	−0.738**	−0.811**	−0.670*	−0.878***	−0.784**	−0.837***
AOPP	–	–	+ 0.844**	+ 0.784**	+ 0.656*	–	+ 0.611^†^	+ 0.535^†^	+ 0.544^†^
PA	–	–	+ 0.660^†^	–	–	–	–	–	–
APA	–	–	+ 0.987***	+ 0.873***	+ 0.580^†^	–	+ 0.578^†^	–	+ 0.528^†^
TP	–	–	+ 0.876***	+ 0.730*	–	–	–	–	–

FRAP: ferric reducing ability of plasma; TAC: total antioxidant capacity; FRSA: free radical scavenging activity; TTL: total thiol levels; TOS: total oxidant status; NOx: nitric oxide metabolites; AOPP: advanced oxidation protein products; PA: protease activity; APA: antiprotease activity; TP: total protein; Osm: osmolarity; Vol: volume; Conc: sperm concentration; Prod: sperm production; Tot mot: total sperm motility; Prog: progressive sperm motility; VCL: curvilinear velocity; VSL: straight-line velocity; VAP: average path velocity. *, **, ***: indicate a statistic significance for *P* < 0.05; *P* < 0.01 and *P* < 0.001, respectively; ^†^: indicate a tendency for *P* < 0.10.

## Discussion

The oxidative profile and the protease/protease inhibitor activities of SP were related to sperm functionality in donkey stallions. Interesting correlations emerged both between FRAP, TAC, and NO_x_ with most of the seminal parameters and sperm kinetics as well as between most of the analyzed parameters when related to each other. The azoospermic stallion showed, together with a lower seminal volume and TP and APA, a lower antioxidant status when measured with FRAP, TTL, and TAC assays as well as higher NO_x_ and AOPP values in comparison with the normospermic stallions. It seems important to point out that the azoospermic stallion was affected by a form of non-obstructive azoospermia; this condition can be discriminated from obstructive azoospermia based on the alkaline phosphatase (AP) content in SP^27^. In our study, the AP content in azoospermic stallion did not differ from that of the normospermic stallions (193.2 ± 77.7 vs*.* 153.3 ± 90.4 µkat/L in the azoo- and normo-spermic stallions, respectively) (data not shown). Therefore, the differences in the oxidative profile and in the proteolysis regulators discriminating the azoospermic individual from the normospermic stallions cannot be addressed to the lack of the testicular and epididymal fluid contribution to the SP.

Recently, the SP antioxidant status, measured by the activities of four antioxidant enzymes (superoxide dismutase, catalase, glutathione peroxidase, glutathione reductase), was related to sperm functionality in horse and donkey stallions^28^. In this study, SP antioxidant capacity was significantly higher in donkey than in horse stallions, and the activities of superoxide dismutase and catalase in donkeys positively correlated with some sperm kinematic parameters, as progressive motility, linearity, and straightness; however, no correlation was found in horse stallions^28^. The higher enzymatic activities in donkey SP might be related to the higher functional activity of the accessory glands in donkey than in horse stallions^29^. Moreover, analyzing blood serum and SP in azoo- and normo-spermic men, Gulum and colleagues^30^ found higher levels of oxidative parameters and lower levels of antioxidant parameters in the azoospermic group. In the present paper, the SP antioxidant status was assessed by different methods whose analytical results are the expression of the cumulative actions of all the antioxidants present in this biological matrix, thus providing an integrated parameter rather than the sum of the single measurable substances. Among these assays, FRAP and TAC positively correlated with most of the analyzed parameters of sperm functionality. FRAP reflects the effect of low molecular weight antioxidants, mainly measuring uric acid, α-tocopherol, bilirubin, and ascorbic acid levels, rather than antioxidants containing sulfhydryl groups in their structure, as glutathione (GSH) and albumin. In contrast, TAC assay also measures albumin, whose proteoforms correlated with stallion sperm motility parameters^31^. Moreover, the uric acid, whose concentration is higher in high fertility stallions^32^, affects the ABTS-based method less than the FRAP assay^33,34^. All these assays are widely used in fertility diagnostics in men^35–38^; however, recently, FRAP and TAC assays have been applied to bovine SP and sperm lysates and positively correlated with sperm motility parameters^39^.

Alternative methodological approaches for evaluating SP antioxidant activity are FRSA and TTL. They positively correlated with sperm concentration and production; however, they did not correlate with sperm kinematic parameters. Lower FRSA and TTL levels were found in the azoospermic stallion in agreement with previous studies in human sperm^40^ showing a lower content of thiol groups in infertile than control patients. TTL assay is a little-used method for the assessment of semen quality in livestock species. Notwithstanding in our study TTL only correlated with sperm concentration and production, we believe it represents a valid tool for sperm quality assessment based on the pivotal role played by thiols in protecting spermatozoa from oxidative damage^41^. This is supported by a previous study in stallions showing that low TTL and ABTS-based assay values in SP were associated with a massive occurrence of sperm DNA breaks^42^.

Since reactive oxygen species (ROS) and reactive nitrogen species (RNS) are highly unstable and reactive, an approach for evaluating the effects of oxidative and nitrosative stress on biological matrices consists in assessing more stable and reliable derived-ROS and -RNS compounds by simple spectrophotometric assays that can be automated on clinical auto-analyzers allowing rapid and inexpensive data collections. Nitric oxide (NO) is a free radical widely involved in many biological processes, including reproduction. It is very difficult to detect and, hence, it is indirectly evaluated through its metabolites^43^. The role of NO in sperm physiology is still unclear although it is well-known that it is involved in sperm motility, capacitation, and fertilization in mammals^44,45^. A recent study demonstrated that elevated NO metabolites were associated with sperm dysfunction in horse stallions^46^. Our results showed higher NO_x_ levels in the azoospermic individual than in the normospermic stallions and significant negative correlations with all sperm function parameters. These findings are in agreement with studies in humans, where NO metabolite levels, analyzed as nitrite (NO_2_^−^) concentration, were statistically higher in infertile men than in controls; besides, the NO_2_^−^ concentration negatively correlated with sperm motility^47^.

TOS assay evaluates the total oxidant substances present in biological fluids^48^. In our study, this test did not correlate with sperm function but it correlated with ejaculate volume. Based on these findings, TOS does not seem a valid predictor of sperm functionality in donkeys. In contrast, in human semen, higher TOS values were associated with sperm motility impairment and lower total antioxidant status, assessed by ABTS-based test; in this study, TOS and TAC values were negatively correlated^49^. Also, Verit et al.^50^, comparing fertile and idiopathic sub-fertile men, found TOS negatively correlated with sperm concentration, motility, and morphology.

AOPP assay evaluates the oxidative protein modifications by detecting dityrosine-containing protein cross-linked products. This analysis was established to assess proteins, mostly aggregates of albumin, in blood plasma^51^; however, it was efficiently applied in other organic matrices as SP^37^. In our study, the AOPP levels were positively correlated with some descriptive parameters of sperm function, as sperm concentration, production, and total motility. Moreover, AOPP levels were not detectable in the SP of the azoospermic stallion. This finding may depend on the lower total protein content found in the azoospermic individual with respect to the normospermic samples, ponying the detection below its analytical limit. The consequences of protein oxidation on reproductive function in mammals are still unclear. In human SP, AOPP levels were negatively related to fertility, with higher concentrations in azoo- and terato- in comparison to normo-spermic individuals^37^. Similarly, when protein oxidation level of SP was measured as carbonyl content, its concentration was higher in infertile men^52^. On the contrary, in stallion spermatozoa, carbonyl content was positively related to motility and viability, with a higher level of oxidized sperm proteins in fertile animals during the breeding season^53^. This occurrence, however, was not associated with sperm DNA damage^53^ and could be part of that paradoxical effect between free radical production and sperm function found in stallion sperm by Aitken and colleagues^54^. On the other hand, SP protein oxidation has been related to several defects in sperm function of sub-fertile stallions during the non-breeding season^53^.

Among the constituents of mammalian SP, a complex of proteins and enzymes with protease and protease inhibitor activities, namely proteolysis regulators, plays a role in the reproductive biology that is still poorly known. In humans, proteolysis regulators affect sperm maturation, activation, and storage as well as semen coagulation and liquefaction. SP protease inhibitors are further involved in sperm protection from proteolytic enzymes released from damaged/dead spermatozoa^26^. Proteolysis regulators seem to be involved in fertilization, allowing the spermatozoon to penetrate both the zona pellucida and oocyte plasma membrane^55^. For an overall assessment of these activities in donkey SP, we applied two simple and reproducible spectrophotometric methods, previously used to assess the proteolysis regulator activities in fish^56^, rather than proteomics approaches already experienced in mammals^26^. In our study, both protease and antiprotease activities correlated with many of the other analyzed SP parameters. Moreover, antiprotease activity strongly correlated with sperm concentration and motility (*P* < 0.001). This fits with results obtained in some fish species, showing a strong correlation between antiprotease activity and sperm concentration^57^, and in humans, where protease inhibitors are involved in sperm capacitation and motility and are associated with semen fertility^26,58^. All this suggests that the assessment of the activity of proteolysis regulators represents a useful tool for obtaining more information on seminal quality.

In conclusions, the analysis of the oxidative profile and the activity of proteolysis regulators in donkey seminal plasma showed that most of the results obtained by the different methods used were well correlated with each other. However, only some of them, as FRAP, TAC, and NO_x_, were significantly correlated with seminal characteristics and sperm kinetics. The comparison carried out between different methods was aimed at evaluating the oxidative profile of an organic matrix such as seminal plasma and did not intend to define selection criteria among assays, considering that each test interrogated different groups of substances. Instead, the correspondence between each assay and sperm function allowed to identify effective tools for assessing semen quality in the donkey species. Preliminary discriminant variations emerged between an azoospermic individual and the normospermic stallions in the antioxidant activity, based on FRAP, TAC, and TTL assays as well as in the nitric oxide metabolites, based on the nitrite/nitrate (NO_x_) levels. Also, the anti-protease activity seems to differ between the azoospermic individual and the normospermic stallions. These differences should be reanalyzed following an increase in the number of cases and, if confirmed, the physiological mechanism underlining them should be investigated.

## Materials and methods

### Reagents

All reagents and media were purchased from Merck (Milan, Italy) unless otherwise stated and cell culture tested.

### Animals

Nine adult and reproductively mature Martina Franca (MF) donkey stallions, aging from 2 to 18 years and weighing from 150 to 450 kg, and one adult and reproductively mature Romanian (RO) donkey stallion, 11 years old and weighing 185 kg, were enrolled in this study. All stallions were trained for semen collection and used in AI programs except one of the nine MF stallions that was affected by non-obstructive azoospermia^27^. Seven MF donkey stallions (one azoo- and six normo-spermic stallions) were housed at the Rustic Found of Chiareto, Veterinary Faculty, University of Teramo (Italy). The remaining three donkey stallions (2 MF and 1 R) were housed at a private farm in the Potenza district (Italy). All animals used were client-owned and informed consent was obtained from the owners. All the donkeys were kept in box stalls with an open paddock, under natural light conditions.

### Ethics approval

This study has been conducted according to the guidelines of the European Directive 63/2010 on the protection of animals used for scientific purposes, transposed into the Italian law by Legislative Decree 2014/26, and followed the ARRIVE guidelines. Considering that the proposed experimental design does not fall within the European Directive 63/2010, the Ethics Committee of the University of Teramo has established that it does not require any authorization for its performance.

### Experimental design

Stallions previously trained for semen collection were subjected to repeated twice weekly semen collections to empty their epididymal reservoir and to grossly assess their semen quality (data not shown). Subsequently, after 4 d from the last preliminary collections, a single ejaculate for each stallion was collected and analyzed in this study. However, in the azoospermic stallion, the semen collection was repeated after one month and both ejaculates were analyzed. SP of each stallion was analyzed for evaluating its oxidative profile by using several methodologies as well as its proteolysis regulator potential. The experimental blinding was assured through an independent assignment of tasks.

### Semen collection and preliminary evaluations

Semen was collected from March to June, as previously described^59^. Semen was filtered by a sterile gauze; the volume was measured by a cylinder and sperm concentration was estimated by a Makler chamber. Sperm production was obtained by sperm concentration × semen volume. A homogeneous part of the semen was centrifuged (2000*g* for 10 min) to separate the SP from cells and debris. SP osmolarity was measured using an osmometer (Digital Osmometer, Roebling, Berlin, DE) and pH was measured a Jenway 3020 pH meter (Jenway, London, UK). SP aliquots of each stallion were stored at −80 °C until analysis.

### Sperm kinetics

Sperm kinetics were evaluated with either SCA 5.0 (Microptic, Barcelona, Spain)^60^ or CASA IVOS 12.3 (Hamilton Thorne Biosciences, Beverly, MA, USA)^61^ systems. Both systems operated at 25 video frames per sec. Six fields per sample were examined (two drops per sample and three fields per drop), and a minimum of 400 spermatozoa were recorded per ejaculate. Before the beginning of the experiment, both systems were compared by cross-checking with frozen semen and properly calibrated. Semen was diluted in INRA96^59^ to a concentration of 20–30 × 10^6^ spermatozoa/mL and samples were equilibrated for 2 min at 37 °C. Spermatozoa with an average velocity of less than 10 µm/s were considered static. Sperm kinetics included: the percentage of motile spermatozoa (Tot Mot); the percentage of progressive spermatozoa (Prog, average path velocity higher than 70 µm/s and straightness of track higher than 80%); the curvilinear velocity (VCL, µm/s); the straight-line velocity (VSL, µm/s); and the average path velocity (VAP, µm/s)^59^.

### Ferric reducing ability of plasma (FRAP)

FRAP assay was performed following the original method of Benzie and Strain^34^. In brief, sodium acetate buffer (pH 3.6), tris(2-pyridyl)-s-triazine (TPTZ), and iron(III) chloride hexahydrate were mixed to generate a FRAP fresh prepared solution. Later, 10 µL of SP samples in duplicates were added to 300 µL FRAP solution and, after 5 min of incubation at 37 °C, the optical densities (ODs) of the reaction mixture were read at 600 nm in a microplate reader (Model 550, BioRad, Segrate, Milan, Italy). The assay was calibrated with iron sulfate heptahydrate (FeSO_4_·7H_2_O) and the results were reported as FeSO_4_·7H_2_O equivalents (μM).

### Total antioxidant capacity (TAC)

TAC, based on the reduction of colored 2,2′-azinobis-(3-ethylbenzothiazoline-6-sulfonic acid) radical cation (ABTS·^+^), was measured according to Erel^33^. Briefly, 5 μL of the SP samples were mixed with 200 μl of acetic acid–sodium acetate buffer (pH 5.8) and measured at 660 nm with the microplate reader. Thereafter, 20 μL of acetic acid–sodium acetate buffer (pH = 3.6) containing 2 mM H_2_O_2_ and 10 mM ABTS were added to the mixtures. After 5 min of incubation at room temperature, the ODs were read again at 660 nm. The assay was calibrated with ascorbic acid (AA) and the results are presented as AA equivalents (μM).

### Free radical scavenging activity (FRSA)

FRSA was analyzed by the 2,2-Di(4-tert-octylphenyl) -1-picrylhydrazyl (DPPH) reduction assay, based on the reduction of DPPH· to 1,1-diphenyl -2-picryl hydrazine, as described by Blois^62^ with minor modifications^63^. In brief, 25 µL of the SP samples were mixed with 475 µL of 10 mM PBS, pH 7.4, and 500 µL of a 0.1 mM DPPH diluted in methanol. The mixture was kept for 30 min in darkness at room temperature, before absorbance reading at 520 nm against the blank, using a spectrophotometer (SmartSpec 3000 UV/Vis, Bio-Rad, Segrate, Italy). The absorbance of each sample was related to DPPH in PBS solution. The decrease of DPPH bleeding was calculated as follows: % of inhibition = [1 − (As/A0)] × 100; where As is the absorbance of the sample and A0 is the absorbance of the DPPH solution.

### Total thiol levels (TTL)

TTL (sulfhydryl group, –SH) were measured as indicated by Hu^64^. Thiols interact with 5,5 -dithiobis-(2-nitrobenzoic acid) (DTNB), and form a highly colored anion with the highest peak at 412 nm (ε412 = 13,600 M^−1^ cm^−1^). In brief, 50 µL of the SP samples were mixed with 1 mL of Tris–EDTA buffer (0.25 M Tris base, 20 mM EDTA, pH 8.2), and the ODs were read at 412 nm against blank in the spectrophotometer. Next, 20 µL of 10 mM DTNB in absolute methanol were added to the solutions. After 15 min at ambient temperature, the ODs were read again against the DTNB blank. TTL were expressed in µM.

### Total oxidant status (TOS)

TOS was measured according to Erel^48^, based on the oxidation of ferrous ion to ferric ion and assessed by xylenol orange. In brief, 35 µL of the SP samples were mixed with 225 µL of 25 mM H_2_SO_4_ solution (pH 1.75) containing 150 mM xylenol orange, 140 mM NaCl, and 1.35 M glycerol, and read with the microplate reader at 560 nm. Thereafter, 11 μl of 25 mM H_2_SO_4_ solution containing 5 mM ammonium iron (II) sulfate and 10 mM *o*-dianisidine dihydrochloride were added to the mixtures. After 5 min of incubation at room temperature, the ODs were read again at 560 nm. The assay was calibrated with *tert*-butyl hydroperoxide (*t*-BHP) and the results were presented as *t*-BHP equivalents (μM).

### Nitrite/nitrate (NO_x_) levels

Nitric oxide radical (NO·) was measured quantifying its stable metabolites (NO_x_), namely the sum of nitrite (NO_2_^−^) and nitrate (NO_3_^−^)^65^. In brief, 100 µL of deproteinized SP samples were treated with 100 µL of 0.8% vanadium (III) chloride in 1 M HCl to reduce nitrate to nitrite. Thereafter, 100 μL of Griess reagent (0.2% sulfanilamide and 0.1% N-1-(naphthyl)ethylenediamine) were added and the mixtures were incubated for 30 min at 30 °C. Finally, the ODs were read at 540 nm in the microplate reader. The assay was calibrated with sodium nitrate (NaNO_3_) and the results were reported as NaNO_3_ equivalents (μM).

### Advanced oxidant products (AOPP)

AOPP was analyzed as Hanasand et al.^66^. Briefly, 200 µl SP samples were added to 800 µl 0.2 M citric acid. Different concentrations of chloramine-T (3.125–100 µM) in citric acid (calibration standards) were added to 10 µL of 1.16 M potassium iodide. After 2 min, ODs were read at 340 nm in the spectrophotometer and the AOPP concentrations were expressed as chloramine-T equivalents (μM).

### Protease activity (PA)

PA of samples was determined using the azocasein hydrolysis assay^67^, with minor changes. Briefly, an equal volume of each SP sample was added to 0.7% azocasein in 100 mM ammonium bicarbonate, pH 7.8. Tubes were placed on a shaker at 30 °C for approximately 19 h. The reaction was stopped by adding trichloroacetic acid (4.5% final concentration). After centrifugation (10,000 g, 15 min), 100 µl of each supernatant in duplicate was added to 100 µl of 0.5 M NaOH and ODs read at 450 nm with the microplate reader. Results are expressed as a percentage of activity in relation to a positive control that was the bovine trypsin.

### Anti-protease activity (APA)

APA was determined by the capacity of the sample to inhibit trypsin activity^68^. In brief, 20 µL of the SP samples were incubated with 5 mg mL^-1^ trypsin solution for 10 min at 22 °C. Subsequently, 200 µL of 0.1 M phosphate buffer solution, pH 7.0, and 250 µL 2% azocasein were added and incubated for 10 min at 22 °C. The reaction was stopped by the addition of 500 µL of 10% trichloroacetic acid and further incubated for 30 min at 22 °C. After centrifugation (8000*g*, 10 min), 100 µL 1 N NaOH was added to 100 µL of supernatants and the ODs were read at 450 nm with the microplate reader. In the positive control, buffer replaced the SP sample whereas, in the negative control, buffer replaced both the SP sample and the trypsin. The percentage of inhibition of trypsin activity by each SP sample was calculated by comparing it to the positive control.

### Total protein (TP) concentration

TP concentrations were measured by the Biuret method using a total protein reagent (T1949). The assay was calibrated with bovine serum albumin (BSA) and the TP concentration is presented as BSA equivalents (mg mL^-1^).

### Statistical analysis

Data from the nine normospermic and one azoospermic stallions entered into a datasheet, as follows: the experimental date, the sampling number, the duplicate number (1–2), the ten SP parameters (i.e., FRAP, TAC, FRSA, TTL, TOS, NO_x_, and AOPP assays as well as TP, PA, and APA), the five semen characteristics (i.e., semen pH, osmolarity and volume as well as sperm concentration and production) and the five sperm kinematic endpoints (i.e., total and progressive motility, VCL, VSL, VAP). These data were analyzed by using Systat 11.0 (SYSTAT Software Inc., San Jose, California, USA).

The Shapiro-Wilks test was used to evaluate the normal data distribution and the Levene's test to evaluate the homogeneity assumption needed for carrying out parametric tests. Variables displaying a not normal distribution, as percentages, were transformed into angles corresponding to arcsine of the square root for variance analyses. The pH values did not follow a continuous distribution, so H^+^ concentrations were log-transformed before the analysis. Coefficients of correlation (R) were calculated by linear regression procedure (SYSTAT 11.0). The minimum level of statistical significance was *P* < 0.05. Values are presented as mean ± standard deviation (SD).
